# Distinct clinical and microbial profiles in left-sided and right-sided colorectal cancer: a comprehensive analysis

**DOI:** 10.1128/spectrum.00336-25

**Published:** 2026-03-13

**Authors:** Jianjiong Li, Buyuan Dong

**Affiliations:** 1 Department of Colorectal and Anal Surgery, The First Affiliated Hospital of Ningbo University 117881, Ningbo, Zhejiang, China; 2 Department of Gastroenterology, Ningbo No.2 Hospital, Wenzhou Medical University, Ningbo, Zhejiang, China; University of Guelph College of Biological Science, Guelph, Ontario, Canada

**Keywords:** colorectal cancer, gut microbiome, microbial diversity, left-sided colorectal cancer, right-sided colorectal cancer

## Abstract

This study investigates the clinical and microbial differences between left-sided colorectal cancer (LCC) and right-sided colorectal cancer (RCC), as well as the microbial profiles in tumor tissues (T) and peritumoral tissues (P) of colorectal cancer (CRC) patients. A total of 240 tissue samples were collected from CRC patients and classified based on the tumor location (LCC vs RCC) and tissue type (tumor vs peritumoral). Clinical data were recorded, and microbial analysis was performed using 16S rRNA sequencing. Bioinformatics analysis assessed microbial diversity and community composition, with statistical tests identifying significant differences between groups. LCC patients were older, heavier, and taller, with higher levels of tumor markers (CEA and CA199). Microbial analysis showed no significant differences in species evenness or richness between LCC and RCC; overall beta-diversity patterns did not differ significantly, although several taxa and predicted functions exhibited differential abundance between the two groups. Functional analysis revealed significant differences in KEGG Orthologs (KOs) and Clusters of Orthologous Groups (COGs) between LCC and RCC. Tumor tissue exhibited lower microbial diversity compared to peritumoral tissue, with significant differences in the microbial community structure. Our findings highlight the importance of considering sidedness and microbial composition in CRC management. The distinct microbial profiles in tumor and peritumoral tissues provide potential biomarkers for early detection and treatment response prediction. Future research should further explore these differences and their clinical implications.

**IMPORTANCE:**

Colorectal cancer (CRC) exhibits marked biological and prognostic differences between left-sided (LCC) and right-sided (RCC) tumors, yet the role of the tumor-associated microbiome stratified by sidedness remains poorly defined. In this study of 240 Chinese patients, we report the first integrated analysis combining clinical features with tissue microbial profiles (tumor vs peritumoral) according to the primary tumor location. We report differential abundance of specific taxa and predicted metabolic functions between LCC and RCC, plus significantly reduced diversity and altered structure in tumor tissue (T) compared with the adjacent normal tissue. These findings underscore the necessity of considering tumor sidedness and intratumoral microbiota in CRC classification, risk assessment, and precision therapy and highlight candidate microbial signatures as potential noninvasive biomarkers for early detection, sidedness prediction, and personalized treatment strategies.

## INTRODUCTION

Colorectal cancer (CRC) is a significant global health issue, ranking as one of the most commonly diagnosed cancers and a leading cause of cancer-related mortality. In 2020, approximately 1.9 million new cases of CRC were reported worldwide, with around 935,000 deaths attributed to the disease, highlighting its severe impact on public health (1). The incidence and mortality rates of CRC vary significantly across different regions and are influenced by factors such as socioeconomic status, lifestyle, and dietary habits. For instance, countries with higher Human Development Index (HDI) levels tend to have lower mortality rates, indicating a correlation between socioeconomic factors and cancer outcomes (2). The distinction between left-sided colorectal cancer (LCC) and right-sided colorectal cancer (RCC) is crucial in understanding the disease’s biology and prognosis. Research indicates that RCC is often diagnosed at a more advanced stage compared to LCC, which is associated with better survival outcomes. Specifically, patients with RCC tend to present with higher-grade tumors and more aggressive disease characteristics, leading to poorer overall survival rates. The median cause-specific survival (CSS) for LCC is superior to that of RCC, particularly with the advent of modern chemotherapy regimens, which have further widened the survival gap between these 2 groups (3). Several factors influence the risk and outcomes of colorectal cancer. Age is a significant risk factor, with the majority of cases occurring in individuals aged over 50 years (4). Other critical factors include body mass index (BMI), lifestyle choices such as diet and physical activity, and genetic predispositions. For example, high BMI has been linked to increased mortality from CRC, while dietary factors, including high consumption of red and processed meats, have been associated with elevated cancer risk (5). Additionally, socioeconomic factors, such as access to healthcare and screening programs, play a vital role in determining CRC outcomes. In particular, disparities in screening uptake have been observed among different racial and ethnic groups, with African Americans facing higher mortality rates due to lower screening rates and access to care (6). Moreover, the relationship between CRC and noncancer causes of mortality is increasingly recognized. Studies indicate that cardiovascular diseases are among the leading noncancer causes of death in CRC patients, particularly in the years following diagnosis (7). This highlights the need for comprehensive care that addresses not only cancer treatment but also the management of comorbid conditions.

The gut microbiota, a complex community of microorganisms residing in the gastrointestinal tract, plays a significant role in human health and disease, particularly in the context of CRC. Recent research has highlighted the intricate relationship between gut microbiota and CRC, revealing how dysbiosis—an imbalance in microbial communities—can contribute to the initiation and progression of this malignancy. Studies have shown that specific bacterial populations are associated with CRC. For instance, an increased abundance of certain bacteria, such as *Fusobacterium nucleatum* and *Escherichia coli*, has been linked to cancer development, while other species, like Faecalibacterium prausnitzii, appear to have protective effects against CRC (8). The mechanisms by which gut microbiota influence CRC include modulation of immune responses, production of metabolites that can promote or inhibit tumor growth, and the potential to induce inflammation, which is a known risk factor for cancer (9, 10).

Research has also focused on the differences in gut microbiota between LCC and RCC. These two types of colorectal cancer exhibit distinct biological behaviors, molecular characteristics, and responses to treatment. For example, RCC is often associated with a higher prevalence of microsatellite instability (MSI) and BRAF mutations, while LCC tends to show chromosomal instability (11, 12). The microbiota composition in these two regions of the colon also differs significantly. Studies have indicated that the microbial taxa in left-sided colon samples, such as Clostridium perfringens and *Fusobacterium nucleatum*, are more abundant and may promote tumorigenesis, whereas right-sided colon samples are enriched with Bifidobacterium dentium, which is considered less harmful (12, 13). Recent findings suggest that the gut microbiota’s role in CRC is not only limited to tumor initiation but also extends to influencing treatment outcomes. For instance, the presence of certain microbial species can affect the efficacy of chemotherapy and immunotherapy, highlighting the potential for microbiome-targeted therapies in CRC management (14). Furthermore, the identification of specific microbial signatures associated with different tumor locations may provide valuable biomarkers for predicting disease progression and treatment responses (13).

In conclusion, the gut microbiota plays a crucial role in the pathogenesis of colorectal cancer, with distinct differences observed between right-sided and left-sided colorectal cancers. This study aims to elucidate these differences further and explore the therapeutic potential of modulating the gut microbiota to improve prevention and treatment strategies for colorectal cancer. Understanding the complex interplay between gut microbiota and colorectal cancer is essential for developing personalized cancer therapies.

## MATERIALS AND METHODS

### Sample collection and grouping

A total of 240 tissue samples were collected from CRC patients. Based on the location of tumor growth, the samples were classified into LCC and RCC. According to the tissue type, they were divided into tumor tissues (T) and peritumoral tissues (P). Clinical data for each participant were recorded, including age, gender, CRC stage, and concentrations of tumor markers.

### DNA extraction and amplification

Total genomic DNA was extracted using the MagPure Soil DNA LQ Kit (Magan) following the manufacturer’s instructions. DNA concentration and integrity were measured with NanoDrop 2000 (Thermo Fisher Scientific, USA) and agarose gel electrophoresis. Extracted DNA was stored at −20°C until further processing. The extracted DNA was used as the template for PCR amplification of bacterial 16S rRNA genes with the barcoded primers and Takara Ex Taq (Takara). For bacterial diversity analysis, the V3–V4 variable regions of 16S rRNA genes were amplified with universal primers 343F (5′-TACGGRAGGCAGCAG-3′) and 798R (5′-AGGGTATCTAATCCT-3′) (15).

### Library construction and sequencing

The amplicon quality was visualized using agarose gel electrophoresis. The PCR products were purified with AMPure XP beads (Agencourt) and amplified for another round of PCR. After being purified with the AMPure XP beads again, the final amplicon was quantified using the Qubit dsDNA Assay Kit (Thermo Fisher Scientific, USA). The concentrations were then adjusted for sequencing. Sequencing was performed on an Illumina NovaSeq 6000 with 250 bp paired-end reads (Illumina Inc., San Diego, CA; OE Biotech Company; Shanghai, China).

### Bioinformatic analysis

The library sequencing and data processing were conducted by OE Biotech Co., Ltd. (Shanghai, China). Raw sequencing data were in FASTQ format. Paired-end reads were then preprocessed using Cutadapt software to detect and cut off the adapter. After trimming, paired-end reads were filtered for low-quality sequences, denoised, merged, and detected, and the chimera reads were cut off using DADA2 (16) with the default parameters of QIIME2 (17) (2020.11). At last, the software outputs the representative reads and the ASV abundance table. The representative read of each ASV was selected using the QIIME2 package. All representative reads were annotated and blasted against the Silva database (Version 138) using a q2-feature-classifier with default parameters.

### Statistical analysis

QIIME2 software was used for alpha and beta diversity analyses. The microbial diversity in samples was estimated using the alpha diversity that includes the Chao1 index (18) and Shannon index (19). The weighted UniFrac distance matrix performed by the R package was used for weighted UniFrac principal coordinates analysis (PCoA) to estimate the beta diversity. Then, the R package was used to analyze significant differences between different groups using ANOVA/Kruskal-Wallis/T test/Wilcoxon statistical test. The linear discriminant analysis effect size (LEfSe) method was used to compare the taxonomy abundance spectrum.

## RESULTS

### Clinical characteristics of CRC population by the sidedness status

There are 176 left-sided and 64 right-sided colorectal cancer patients. The average age of left-sided patients is 66.6 years, which is significantly higher than that of right-sided patients at 62.6 years (*P* = 0.005). Left-sided patients also have a higher average weight of 66.2 kg compared to 61.7 kg for right-sided patients, with a significant difference (*P* = 0.004). In terms of height, left-sided patients are taller on average at 165.9 cm, while right-sided patients have an average height of 162.6 cm, and this difference is statistically significant (*P* = 0.005). Regarding tumor markers, left-sided patients exhibit higher median levels of CEA (3.2 ng/mL) compared to right-sided patients (2.0 ng/mL), with a significant difference (*P* = 0.007). Although the gender distribution shows a slightly higher proportion of females in the right-sided group (56.2%) compared to the left-sided group (42.0%), this difference is not statistically significant (*P* = 0.051). The T-stage distribution differs significantly between the 2 groups (*P* = 0.017), with a higher proportion of stage 3 tumor in the right-sided group. The N-stage and M-stage distributions do not show significant differences (*P* = 0.080 and *P* = 0.444, respectively). The RAS status also differs significantly (*P* < 0.001), with a higher proportion of wild-type RAS in the left-sided group (56.8%) compared to the right-sided group (31.2%). These findings suggest that the sidedness status might influence various patient characteristics and tumor features, except for the gender distribution (Table 1).

**TABLE 1:** Patient characteristics by sidedness status

	Result for:		P-value
	LCC group	RCC group	
*N*	176	64	
Age (yr)	66.6 ± 8.9	62.6 ± 12.0	0.005
Weight (kg)	66.2 ± 10.6	61.7 ± 10.3	0.004
Height (cm)	165.9 ± 8.1	162.6 ± 7.7	0.005
BMI	23.9 ± 3.1	23.2 ± 2.8	0.115
CEA (ng/mL)	3.2 (1.5–7.0)	2.0 (0.9–5.9)	0.007
CA199 (U/mL)	17.7 (8.2–27.5)	12.6 (7.4–17.8)	0.046
CA125 (U/mL)	8.8 (5.9–11.8)	11.9 (7.1–12.9)	0.029
Sex			0.051
Female	74 (42.0%)	36 (56.2%)	
Male	102 (58.0%)	28 (43.8%)	
T stage			0.017
1	2 (1.1%)	0 (0.0%)	
2	10 (5.7%)	0 (0.0%)	
3	96 (54.5%)	48 (75.0%)	
4	68 (38.6%)	16 (25.0%)	
N stage			0.080
0	68 (38.6%)	36 (56.2%)	
1	56 (31.8%)	12 (18.8%)	
2	36 (20.5%)	10 (15.6%)	
**3**	16 (9.1%)	6 (9.4%)	
M stage			0.444
0	154 (87.5%)	54 (84.4%)	
1	18 (10.2%)	10 (15.6%)	
2	2 (1.1%)	0 (0.0%)	
3	2 (1.1%)	0 (0.0%)	
RAS			< 0.001
Wild type	100 (56.8%)	20 (31.2%)	
Mutant	76 (43.2%)	44 (68.8%)	

### Comparison of microbial abundance and diversity in CRC by the sidedness status

**Fig 1:**
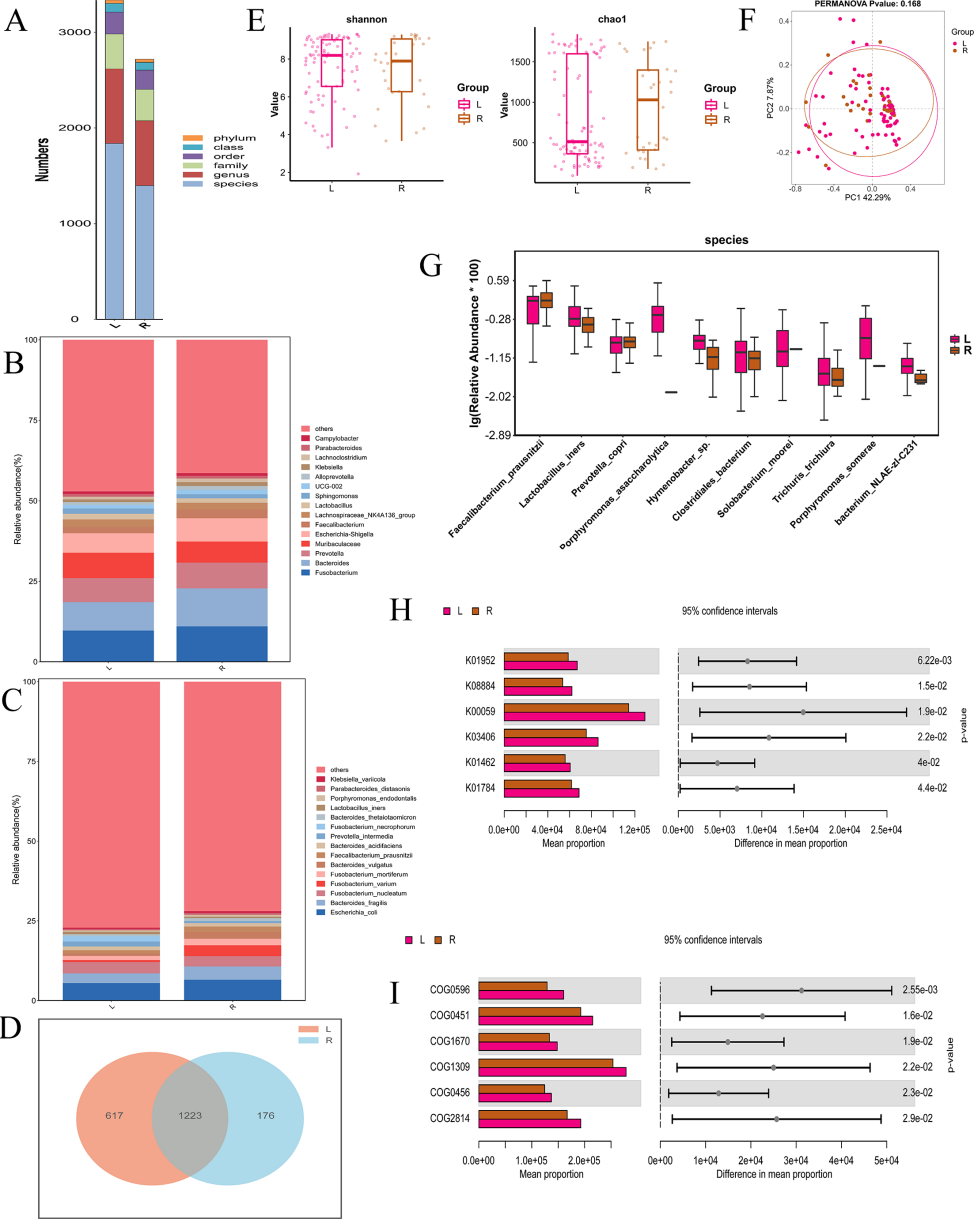
(**A**) Bar chart of the community structure. (**B**) The barplot of the phylum community structure in the two groups. (**C**) The barplot of the species community structure in the two groups. (**D**) Venn diagram. (**E**) The Shannon and Chao1 indices. (**F**) The principal coordinates analysis (PCoA). (**G**) Relative abundance in the top-10 species in the two groups was assessed using appropriate statistical tests. (**H**) KEGG functional classification of the top 15 differential predicted KOs between the two groups. (**I**) COG functional classification of the top 15 differential predicted functions between the two groups. L, left-sided colorectal cancer; R, right-sided colorectal cancer.

At various taxonomic levels including phylum, class, order, family, genus, and species, we analyzed the relative abundance of genus and species in the RCC and LCC groups (Fig. 1A). At the genus level, Fusobacterium was the most abundant, comprising 11.05% vs 9.73%. We also analyzed the relative abundance of species in the RCC and LCC groups. *Escherichia coli* was the most abundant, comprising 6.54% vs 5.51% (Fig. 1B and C). A Venn diagram was used to identify the 1,223 out of 2,016 species shared by the 2 groups (Fig. 1D).

Next, we conducted an alpha diversity analysis on the samples. Boxplot comparison of the Shannon index between LCC and RCC showed no significant difference (*P* > 0.05). Alpha diversity (Shannon and Chao1 indices) did not differ significantly between LCC and RCC (*P* > 0.05), suggesting comparable community evenness and richness between the two groups (Fig. 1E). A 2D PCoA plot using weighted UniFrac distances was generated to compare the microbial community compositions between LCC and RCC. The plot shows the first 2 principal coordinates (PC1 and PC2), explaining 42.29% and 7.87% of the variation, respectively. PCoA based on weighted UniFrac distances did not show a significant separation between LCC and RCC, suggesting broadly similar overall community structures (Fig. 1F). The taxa shown include *Faecalibacterium prausnitzii*, *Lactobacillus iners*, *Prevotella copri*, *Porphyromonas asaccharolytica*, and other top-ranked taxa (Fig. 1G). Figure 1H shows a bar chart comparing the mean proportions of 6 KOs (K01952, K08884, K00059, K03406, K01462, and K01784) between LCC and RCC, with significant differences indicated by *P*-values (Fig. 1H). Fig. 1I presents a bar chart comparing the mean proportions of 6 COGs (COG2814, COG0456, COG1309, COG1670, COG0451, and COG0596) between Group L and Group R. The chart indicates significant differences between the groups, with all *P*-values less than 0.05 (Fig. 1I).

### Comparison of microbial abundance and diversity between the tumor tissue and peritumoral tissue

**Fig 2:**
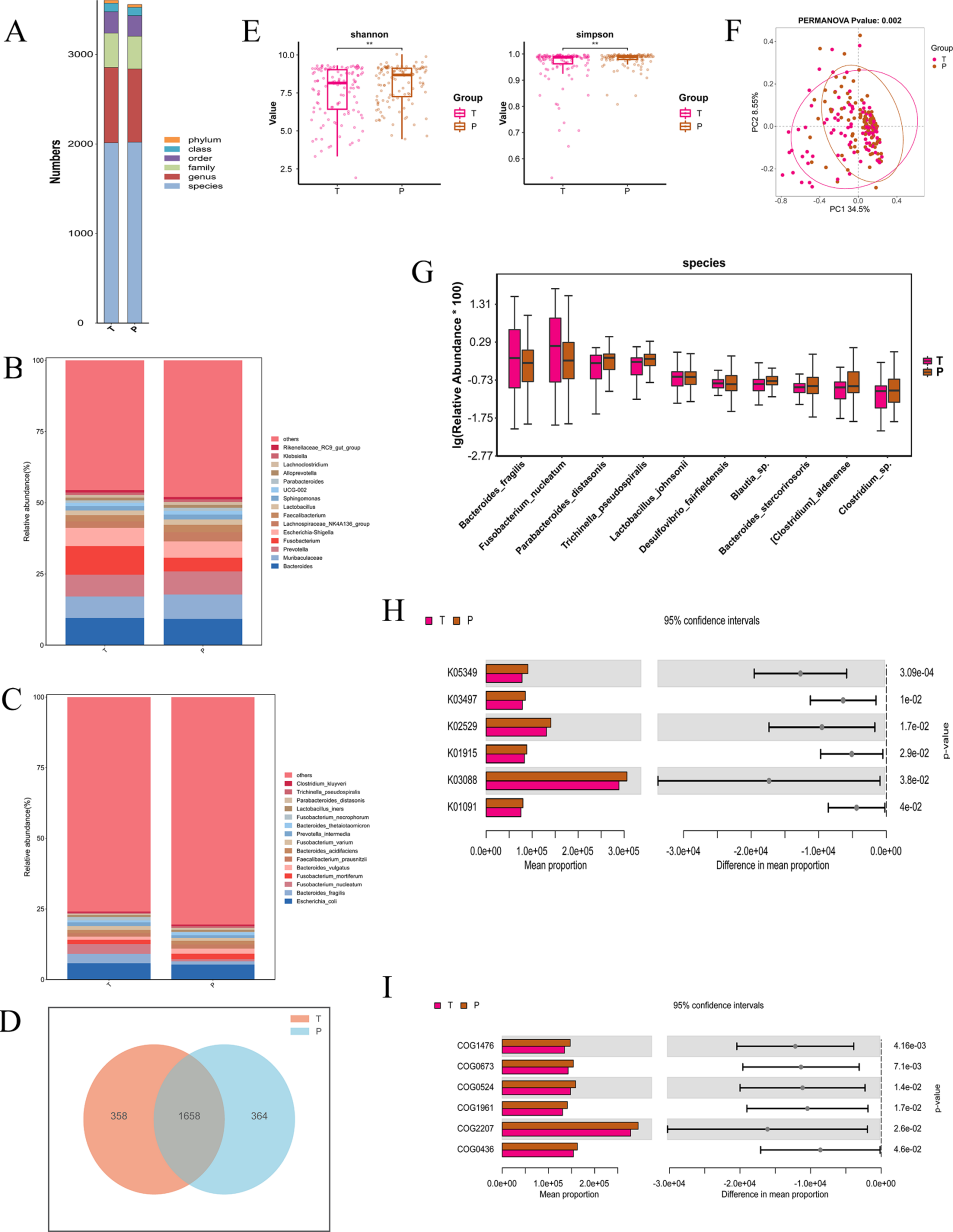
(**A**) Bar chart of the community structure. (**B**) The barplot of the phylum community structure in the two groups. (**C**) The barplot of the species community structure in the two groups. (**D**) Venn diagram. (**E**) The Shannon and Simpson indices. (**F**) The principal coordinates analysis (PCoA). (**G**) Relative abundance in the top-10 species in the two groups was assessed using appropriate statistical tests. (**H**) KEGG functional classification of the top 15 differential predicted KOs between the two groups. (**I**) COG functional classification of the top 15 differential predicted functions between the two groups. T, tumor tissues. P, peritumoral tissues.

At various taxonomic levels including phylum, class, order, family, genus, and species, we analyzed the relative abundance of genus and species in the tumor tissue and peritumoral tissue groups (Fig. 2A). At the genus level, Bacteroides was the most abundant, comprising 9.56% vs 9.24%. We also analyzed the relative abundance of species in the tumor tissue and peritumoral tissue groups, where *Escherichia coli* was the most abundant, comprising 5.79% vs 5.29% (Fig. 2B and C). A Venn diagram was used to identify the 1,658 out of 2,380 species shared by the 2 groups (Fig. 2D).

Next, we conducted an alpha diversity analysis on the samples. Fig. 2E depicts significant differences in the Shannon and Simpson indices between the tumor tissue and peritumoral tissue, indicating higher species diversity and evenness in the peritumoral tissue than in the tumor tissue, as evidenced by the higher median values for both indices in the peritumoral tissue (Fig. 2E). A 2D PCoA plot using weighted UniFrac distances compares the microbial community structures between the tumor tissue and peritumoral tissue, revealing a significant difference (PERMANOVA *P*-value: 0.002). PC1 and PC2 explain 34.5% and 8.55% of the variation, respectively (Fig. 2F). This indicates distinct microbial community compositions between the 2 groups, suggesting different ecological or functional characteristics. Figure 2G shows a boxplot comparing the top 10 differential taxa between tumor and peritumoral tissues, highlighting variations in their log-transformed relative abundances (Fig. 2G). Bar charts compare the mean proportions of 6 KOs (K01091, K03088, K01915, K02529, K03497, and K05349) and 6 COGs (COG0436, COG2207, COG1961, COG0524, COG0673, and COG1476) between tumor tissue and peritumoral tissue, with 95% confidence intervals (Tables S1 and S2). The charts show significant differences between the groups, with *P* < 0.05 (Fig. 2H and I).

## DISCUSSION

The distinction between left-sided colorectal cancer (LCC) and right-sided colorectal cancer (RCC) is crucial for understanding the biology and prognosis of colorectal cancer (CRC). Our study found significant differences in clinical characteristics between LCC and RCC patients. LCC patients were older, heavier, and taller and exhibited higher median levels of tumor markers such as CEA and CA199. Previous studies have shown that left-sided colorectal cancers tend to present with more advanced symptoms, such as hematochezia (the passage of fresh blood through the anus) and narrow stools, which are less common in right-sided cancers. Conversely, right-sided tumors are often associated with more subtle symptoms, leading to later diagnoses and potentially poorer prognoses (20). Accumulating evidence suggests that the pronounced differences in RAS mutational status between left-sided (LCC) and right-sided colorectal cancer (RCC) are mediated, at least in part, by metabolic reprogramming and remodeling of the tumor immune microenvironment, with metabolic status and serum CA199 levels serving as accessible clinical reflections of these processes. In the current cohort, LCC patients exhibited significantly higher serum CA199, and a greater proportion of RAS wild-type tumors—findings that align closely with those of multiple large-scale cohorts and meta-analyses (21, 22). Obesity-associated chronic low-grade inflammation drives hyperactivation of the insulin–IGF-1–PI3K–Akt–mTOR pathway, thereby promoting chromosomal instability (CIN), the predominant oncogenic mechanism in RAS wild-type tumors, in contrast to the microsatellite instability (MSI) or CpG island methylator phenotype (CIMP) pathways more commonly observed in RAS-mutated cancers (23). Concurrently, obesity-induced dysregulation of lipid and bile acid metabolism upregulates sialyltransferase activity and secondary bile acid pools, leading to aberrant glycosylation and increased secretion of sialylated Lewis antigens—the principal epitopes detected by the CA199 assay. This mechanism accounts for the consistently higher CA199 levels observed in LCC (24). Conversely, the markedly higher prevalence of RAS mutations in RCC (68.8% vs 43.2% in LCC) is intimately linked to a profoundly immunosuppressive microenvironment. Oncogenic RAS signaling activates IL-6/STAT3 and TGF-β pathways, inhibits dendritic cell maturation, and recruits regulatory T cells, thereby attenuating antitumor immunity (25). This immunosuppressive profile frequently coexists with MSI-H and BRAFV600E mutations in RCC, molecular features typically seen in patients with lower BMI and normal or only mildly elevated CA199 (26). Thus, BMI and serum CA199 should be regarded not merely as prognostic factors but as integrative “phenotypic readouts” of the complex interplay among tumor sidedness, RAS mutational status, metabolic reprogramming, and immune contexture. Our analysis of the gut microbiota in LCC and RCC revealed no significant differences in species evenness (Shannon index) or richness (Chao1 index) between the two groups. However, several taxa showed differential relative abundances between LCC and RCC. Notably, *Fusobacterium* and *Escherichia coli* were more abundant in RCC, while species such as Porphyromonas asaccharolytica and Lactobacillus iners were more prevalent in LCC. Previous studies have shown that specific bacterial species, including certain strains of Escherichia coli, are more prevalent in left-sided colorectal cancer. For example, *Escherichia coli* is associated with the pathogenesis of colorectal cancer, particularly in left-sided tumors, where it may lead to inflammation and tumorigenesis (11). Moreover, the dysbiosis observed in CRC patients, characterized by an imbalance in gut microbiota, is more pronounced in left-sided tumors. This dysbiosis is often linked to the presence of pathogenic bacteria, including *E. coli*, which can produce toxins and promote inflammation, thereby facilitating tumor development (27, 28).

The functional analysis of the microbial communities revealed significant differences in the abundance of specific KEGG Orthologs (KOs) and Clusters of Orthologous Groups (COGs) between LCC and RCC. For example, KOs such as K01952 (purL, phosphoribosylformylglycinamidine synthase) and K01784 (galE, UDP-glucose 4-epimerase) were more abundant in LCC, suggesting potential roles in carbohydrate metabolism and cell wall biosynthesis (Table S1). LCC had higher levels of K08884 (serine/threonine protein kinase) and K03406 (methyl-accepting chemotaxis protein), which are involved in signal transduction and chemotaxis. The serine-threonine protein kinase Akt, also known as protein kinase B, is a key component of the PI3K-Akt-mTOR signaling pathway, which is frequently deregulated in human tumors. Inhibitors targeting the Akt pathway have been developed, highlighting the potential of this kinase as a therapeutic target in cancer treatment (29). These functional differences may reflect distinct metabolic and signaling pathways active in LCC and RCC, influencing tumor biology and progression.

Similarly, the COG analysis showed significant differences between LCC and RCC. For instance, COG2814 (arabinose efflux permease) and COG1309 (DNA-binding transcriptional regulator) were more abundant in LCC, indicating potential roles in sugar transport and transcriptional regulation. LCC had higher levels of COG0456 (ribosomal protein S18 acetylase) and COG1961 (site-specific DNA recombinase), suggesting involvement in protein synthesis and DNA rearrangement (Table S2). These functional differences highlight the distinct microbial profiles associated with LCC and RCC, which may contribute to their unique biological behaviors.

Comparing the tumor tissue and peritumoral tissue, we found significant differences in microbial diversity and community structure. The peritumoral tissue exhibited higher species diversity and evenness, as indicated by the higher median values of the Shannon and Simpson indices. This suggests that the microbial environment in the peritumoral tissue may be more complex and potentially more conducive to maintaining homeostasis. Previous studies have shown that the Shannon and Simpson indices of peritumoral tissues are slightly higher than those of tumor tissues. Still, there is no significant statistical difference, which may be related to the smaller sample size (30). As shown by the PERMANOVA analysis (*P*-value: 0.002), the significant differences in microbial community structure highlight distinct ecological or functional characteristics between the two tissue types.

The top 10 differential species, including Bacteroides fragilis, *Fusobacterium nucleatum*, and Parabacteroides distasonis, are likely to play key roles in the tumor microenvironment. We conducted statistical analyses on the KEGG and COG results and selected the top 15 most abundant differentially abundant predicted functions (KEGG KOs and COGs) for further analysis. For example, KOs that are more abundant in peritumoral tissue include K01091 (phosphoglycolate phosphatase) and K01915 (glutamine synthetase), suggesting their potential roles in phosphate metabolism and nitrogen fixation. COGs that are more prevalent in peritumoral tissue include COG0436 (aspartate/methionine/tyrosine aminotransferase) and COG2207 (AraC-type DNA-binding domain), indicating their involvement in amino acid metabolism and transcriptional regulation. Previous studies have demonstrated that the gut microbiota is involved in metabolic functions such as energy metabolism, amino acid synthesis, and short-chain fatty acid production, which are crucial for maintaining intestinal health and preventing inflammation (31).

While our study provides valuable insights into the role of gut microbiota in CRC, there are limitations. The sample size may not be large enough to capture all microbial variations, and further studies with larger cohorts are needed to validate our findings. Additionally, longitudinal studies are required to understand the dynamic changes in the gut microbiota during CRC progression and treatment. Future research should also explore the functional roles of specific microbial species and their interactions with host factors, which could lead to the development of novel diagnostic and therapeutic strategies.

Despite these limitations, our study has several strengths. The comprehensive analysis of both clinical and microbial data provides a holistic view of the differences between LCC and RCC. The use of advanced sequencing and bioinformatics techniques ensures the accuracy and reliability of our microbial data. Additionally, the identification of significant differences in microbial profiles between tumor and peritumoral tissues offers new avenues for biomarker development and personalized treatment strategies.

The findings from our study have several implications for the management of CRC. First, the differences in clinical characteristics and microbial profiles between LCC and RCC suggest that a one-size-fits-all approach to diagnosis and treatment may not be optimal. Personalized medicine strategies that consider sidedness and microbial composition could improve patient outcomes. Second, the distinct microbial profiles in tumor and peritumoral tissues provide potential biomarkers for early detection, prognosis, and treatment response prediction. Targeting specific microbial species or pathways could offer new therapeutic avenues for CRC.

### Conclusion

In conclusion, our study highlights the importance of considering sidedness and microbial composition in the management of CRC. Understanding the complex interplay between the gut microbiota and CRC is essential for developing personalized cancer therapies that can improve patient outcomes. The marked enrichment of Fusobacterium in tumor tissue, combined with elevated serum CA199, represents a promising noninvasive biomarker panel for early detection and risk stratification, particularly in left-sided/RAS wild-type CRC. Enrichment of *Fusobacterium nucleatum* in tumor tissue combined with elevated serum CA199 warrants prospective evaluation as a composite risk marker, particularly in Asian populations where such integrated analyses remain scarce.

## Data Availability

The data set for this study is available upon request from the corresponding author.

## SUPPLEMENTAL MATERIAL

Table S1 spectrum.00336-25-s0001.docx KEGG functional description.

Table S2 spectrum.00336-25-s0002.docx COG functional description.

ASM does not own the copyrights to Supplemental Material that may be linked to, or accessed through, an article. The authors have granted ASM a non-exclusive, world-wide license to publish the Supplemental Material files. Please contact the corresponding author directly for reuse.
