# Supplementary material for: Distinct clinical and microbial profiles in left-sided and right-sided colorectal cancer: a comprehensive analysis
Source: Microbiol Spectr. 2026 Mar 13;14(4):e00336-25. doi: 10.1128/spectrum.00336-25 (PMC13055392; doi:10.1128/spectrum.00336-25)
Supplement: Table S1 — KEGG functional description. [file spectrum.00336-25-s0001.docx]

Table.S1 KEGG Functional Description

| Function | Description |
| --- | --- |
| K01952 | purL, PFAS; phosphoribosylformylglycinamidine synthase [EC:6.3.5.3] |
| K08884 | K08884; serine/threonine protein kinase, bacterial [EC:2.7.11.1] |
| K00059 | fabG; 3-oxoacyl-[acyl-carrier protein] reductase [EC:1.1.1.100] |
| K03406 | mcp; methyl-accepting chemotaxis protein |
| K01462 | PDF, def; peptide deformylase [EC:3.5.1.88] |
| K01784 | galE, GALE; UDP-glucose 4-epimerase [EC:5.1.3.2] |
| K01091 | gph; phosphoglycolate phosphatase [EC:3.1.3.18] |
| K03088 | rpoE; RNA polymerase sigma-70 factor, ECF subfamily |
| K01915 | glnA, GLUL; glutamine synthetase [EC:6.3.1.2] |
| K02529 | lacI, galR; LacI family transcriptional regulator |
| K03497 | parB, spo0J; chromosome partitioning protein, ParB family |
| K05349 | bglX; beta-glucosidase [EC:3.2.1.21] |
