# Supplementary material for: Distinct clinical and microbial profiles in left-sided and right-sided colorectal cancer: a comprehensive analysis
Source: Microbiol Spectr. 2026 Mar 13;14(4):e00336-25. doi: 10.1128/spectrum.00336-25 (PMC13055392; doi:10.1128/spectrum.00336-25)
Supplement: Table S2 — COG functional description. [file spectrum.00336-25-s0002.docx]

Table.S2 COG Functional Description

| Function | Description |
| --- | --- |
| COG2814 | Predicted arabinose efflux permease, MFS family |
| COG0456 | Ribosomal protein S18 acetylase RimI and related acetyltransferases |
| COG1309 | DNA-binding transcriptional regulator, AcrR family |
| COG1670 | Protein N-acetyltransferase, RimJ/RimL family |
| COG0451 | Nucleoside-diphosphate-sugar epimerase |
| COG0596 | Pimeloyl-ACP methyl ester carboxylesterase |
| COG0436 | Aspartate/methionine/tyrosine aminotransferase |
| COG2207 | AraC-type DNA-binding domain and AraC-containing proteins |
| COG1961 | Site-specific DNA recombinase related to the DNA invertase Pin |
| COG0524 | Sugar or nucleoside kinase, ribokinase family |
| COG0673 | Predicted dehydrogenase |
| COG1476 | DNA-binding transcriptional regulator, XRE-family HTH domain |
